# Research on Wide-Temperature Rechargeable Sodium-Sulfur Batteries: Features, Challenges and Solutions

**DOI:** 10.3390/ma16124263

**Published:** 2023-06-08

**Authors:** Yimin Liang, Boxuan Zhang, Yiran Shi, Ruyi Jiang, Honghua Zhang

**Affiliations:** 1 Queen Mary University of London Engineering School, Northwestern Polytechnical University, Xi’an 710129, China; 2 School of Chemistry and Chemical Engineering, Northwestern Polytechnical University, Xi’an 710129, China; 3 Institute of Process Engineering, Chinese Academy of Sciences, Beijing 100190, China; 4 Zhengzhou Institute of Emerging Industrial Technology, Zhengzhou 450046, China

**Keywords:** Na-S batteries, wide-temperature, energy density, specific capacity

## Abstract

Sodium-sulfur (Na-S) batteries hold great promise for cutting-edge fields due to their high specific capacity, high energy density and high efficiency of charge and discharge. However, Na-S batteries operating at different temperatures possess a particular reaction mechanism; scrutinizing the optimized working conditions toward enhanced intrinsic activity is highly desirable while facing daunting challenges. This review will conduct a dialectical comparative analysis of Na-S batteries. Due to its performance, there are challenges in the aspects of expenditure, potential safety hazards, environmental issues, service life and shuttle effect; thus, we seek solutions in the electrolyte system, catalysts, anode and cathode materials at intermediate and low temperatures (T < 300 °C) as well as high temperatures (300 °C < T < 350 °C). Nevertheless, we also analyze the latest research progress of these two situations in connection with the concept of sustainable development. Finally, the development prospects of this field are summarized and discussed to look forward to the future of Na-S batteries.

## 1. Introduction

The search for new energy sources has been stimulated by today’s global energy shortages and increased public awareness of caring for the environment [1]. The battery, an important energy storage medium, has the potential to manage and lessen the impact on the environment [2]. Therefore, to meet the demands of burgeoning energy storage applications [3], there is an urgent need to promote the development of systems for rechargeable batteries with low cost [4], long service life and high energy density [5]. Among current battery technologies, due to their advantages of being naturally abundant, having a very high energy density and low cost, Na-S batteries are gaining widespread interest [6].

The Na-S battery story goes back to the 1960s when sodium and sulfur operating in the molten state in the temperature range of 300–350 °C were scheduled and advanced for stationary energy storage owing to their natural abundance and low cost [7], as well as their stability over long periods of cycling [8]. Normally, Na-S batteries operate at high temperatures above 300 °C to maintain the state of the melt of the sulfur cathode and sodium anode [9] and the high ion conductivity of the beta-alumina electrolytes to achieve adequate energy densities and power (Table 1) [10]. However, the high operating temperatures and reactive character of Na and S in the molten state pose significant challenges in terms of lifetime, safety and maintenance, and over the past decade [11], room-temperature Na-S battery systems have been pursued as a promising substitute for both stationary grid storage as well as transport applications [8].

The high theoretical capacity (1672 mA h/g) and abundant resources of sulfur render it an attractive electrode material for the next generation of battery systems [12]. Room-temperature Na-S (RT-Na-S) batteries, due to the availability and high theoretical capacity of both sodium and sulfur [13], are one of the lowest-cost and highest-energy-density systems on the horizon [14]. However, there are still several problems with RT Na-S batteries. These include low sulfur loading, poor rate capability and rapid capacity degradation [15]. The main reasons for these challenges are as follows: (1) the low conductivity of sulfur (5 × 10^−^^28^ S/m) curbs the reaction kinetics [16]; (2) the more severe shuttle effects of soluble polysulfides and side reactions of polysulfides with the carbonate electrolyte lead to low Coulombic efficiency and rapid capacity degradation compared to Li-S and (3) the enormous volume change of sulfur (171%) disrupts the structure of the cathodes, leading to low cycle stability [17].

One of the main challenges of Na-S batteries is the shuttle effect during operation [18]. Polysulfide shuttling effects and slow conversion kinetics of sulfur result in rapid capacity degradation and low capacity, leading to low energy and power densities in practice. The secluding nature of sulfur also exacerbates sluggish conversion reactivity. Various strategies have been reported to conquer the shuttle problem, including the protection of the metallic sodium anode, the development of enhanced electrolytes and advanced sulfur hosts [19]. Since confinement of the sulfur products could basically resolve the shuttle problem, much attention has been paid to research into sulfur host materials. In order to mitigate the shuttle effect, Wang et al. have synthesized S-doped crumpled MXene nanosheets, which exhibit high polarity towards sodium polysulfides [20]. Mo_2_N-W_2_N embedded in a spherical carbon heterostructure with a high level of catalytic activity to convert sodium polysulfides was reported by Yu et al. The reaction kinetics were significantly improved by Xu et al., who composed a three-dimensional rail-like sulfur host formed by carbon nanotubes interconnected with nitrogen-doped porous carbon. However, it is not cost effective to produce sulfur hosts and processes are difficult to scale, contrary to the goal of using low-cost materials and processes for LIBs [21]. Therefore, a key requirement for the development of practical RT-Na-S batteries is the rational design of S-hosts with high sulfur mass loading and efficient conversion kinetics [14].

To sum up, in this review, we will separate Na-S batteries at a wide temperature into two parts and divide them into four parts at different temperatures; then, we will analyze the working mechanism, characteristics, challenges encountered and solutions to provide a cheap and sustainable choice for Na-S batteries [22].

## 2. Na-S Batteries at High Temperature

### 2.1. Reaction Mechanism

**Figure 1:**
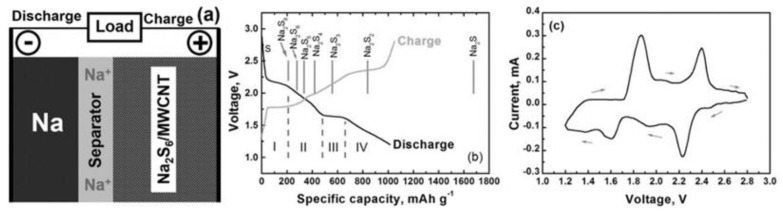
(**a**) Schematic of a Na/dissolved polysulfide cell with an MWCNT fabric electrode, (**b**) Charge/discharge profiles, theoretical versus practical discharge capacities, and (**c**) CV (at a scan rate of 0.1 mV/s) of the Na–S cells operated at ambient temperature. Reproduced with permission [28], Copyright 2014, Chemistry Europe. The Step I, II, III, IV coincide well with the Equations (1)–(6) at P7.

In general, Na-S batteries comprise a sodium-metal anode, an organic liquid electrolyte and a sulfur–carbon composite cathode, as exhibited in Figure 1a. Notably, the exploration of the reaction mechanism of Na-S batteries has continued for decades [23]. It has been proven that the generated Na^+^ ions during the discharge process will pass through the electrolyte and then react with sulfur at high temperatures (300–350 °C) [24], which simultaneously results in the decreases in sulfur on the cathode [25].

The charge and discharge of the Na-S batteries suffered from the complicated process transition processes that contained a series of long-chain (Na_2_S_n_, 4 ≤ n ≤ 8) and short-chain (Na_2_S_n_, 1 ≤ n < 4) sodium polysulfide intermediates. Figure 1b shows the typical charge and discharge profiles of the RT Na-S battery. Besides, the representative cyclic voltammogram of the Na–S battery is also exhibited in Figure 1c [26]. The two dominant reduction peaks in Figure 1b correspond to the two discharge plateaus at ~2.2 V and ~1.6 V [27]. The cathode redox process is highly reversible, as indicated by the two corresponding oxidation peaks during the charging process in Figure 1c [28].

Even though the chemical equations for the basic reaction of the battery are simple as follows: 2Na + S = Na_2_S, the actual reaction is much more complex [29]. In practice, the conductivity of molten sulfur is not ideal, and the researcher typically utilizes a novel strategy to promote the reaction kinetics. In cutting-edge research, most Na-S batteries are based on carbonate electrolytes. In this way, eutectic accelerators become one of the greatest choices, which can greatly improve the electrical conductivity of the material and easily drive the chemical reactions on the electrodes [30].

### 2.2. Features

#### 2.2.1. High Energy Density

The Na-S batteries possess a high energy density of 760 W h/kg [31], which are regarded as one of the highest energy density batteries in the world and thus investigated as an emerging sub-discipline [32]. Despite lithium batteries being utilized as the mainstream of batteries for portable electronic products such as mobile phones, laptops and tablets, the liquid electrolyte of lithium batteries poses safety hazards such as explosions of charging treasures and mobile phones as a consequence of the instability and self-discharge reaction of lithium batteries, retarding their corresponding commercialization. However, high current, high power discharge, no self-discharge reaction, and high charge and discharge current efficiency make Na-S batteries solve the aforementioned issues perfectly [33].

#### 2.2.2. Materials

Except for the advantages of efficacy, sodium batteries also have unique advantages in their materials. The first advantage is that the raw materials (Na, S) utilized to fabricate batteries are plentiful in nature. For instance, a little part concentration of sodium ions in seawater is 1.08 ppm; that is, 1,000,000 g of seawater contains 1.08 g of sodium ions, so a substantial amount of sodium salt can be extracted for a lot of preparation for sodium metal. Then, the acquisition of sulfur, another raw material, is even simpler. Elemental sulfur is mostly found near volcanoes erupting near the seaside, and significant amounts of sulfur can be obtained through a simple mining process. Another advantage of elemental sulfur is that it is non-toxic, which guarantees personal safety when the battery leaks.

### 2.3. Challenges

#### 2.3.1. Expenditure

One of the most significant parts of expenditure is material costs. Although nature has a large amount of sodium storage, to prepare sodium elements, it takes a mass of energy to electrolyze molten sodium salts. This creates the phenomenon that the price paid here is much greater than the convenience of using Na-S batteries [34].

#### 2.3.2. Safety Hazards

The high operating temperatures (300–350 °C) of Na-S batteries implicit harsh safety hazards and may cause supplementary maintenance costs [35]. Furthermore, the half-baked sulfur conversion causes a low use ratio (only ≈ 1/3) of the intellectual capacity of sulfur [36].

#### 2.3.3. Environmental Issues

The United States established regulations for recycling Na-S batteries more than a decade ago, but they still have little effect. The leakage of the contents of Na-S batteries still pollutes large amounts of water and land [37].

#### 2.3.4. Instability of Battery Efficiency

The high-temperature conditions of Na-S batteries have the potential to decrease energy density due to inadequate heat dissipation. Additionally, this may cause ceramic electrolyte fractures, leading to battery failure. Therefore, better heat dissipation measures and suitable substrates are required [35].

### 2.4. Solutions

#### 2.4.1. Combining Solar or Wind Power Generation Systems with Batteries

**Figure 2:**
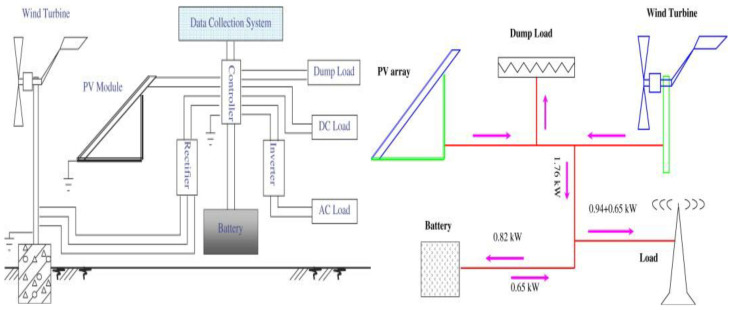
Block diagram of the hybrid solar–wind power generation system and annual average energy balance of the hybrid solar–wind power generation project. Reproduced with permission [41], Copyright 2008, Science Direct.

There are many regions of the world where renewable energy is abundant (Figure 2); for example, Crete Island is full of inexhaustible energy resources like solar and wind. Using wind power to charge Na-S batteries on this island can not only convert many natural resources into electricity and reduce charging costs but also maximize the high conversion rate of Na-S batteries. Molten sodium for the anode and liquid sulfur for the cathode are used in this type of battery [38]. A beta-alumina solid electrolyte separates the positive and negative poles [39]. The technology became commercial in Crete Island, and at the moment, some full-scale plants are in operation in countries such as the US [40]. This method of using reusable natural energy to charge Na-S batteries significantly reduces the costs of battery applications and achieves the goal of reducing operating expenses. Furthermore, the energy provided during the charging process of a Na-S battery is environmentally friendly, which also compensates for the pollution caused by the recycling of these batteries in other aspects.

#### 2.4.2. Adopting More Efficient Thermal Dissipation Measures and Utilizing Electrode Materials with Higher Temperature Tolerance

Because the operating temperature of high-temperature Na-S batteries is not suitable for all materials, such as some reactions with ceramic or graphite electrodes that tend to fail after a long reaction time, we adopt a superior mixed cooling system to prevent thermal runaway. An example of such a system is provided below. Among active cooling, passive cooling, and mixed cooling, the company chose the optimal solution—a passive cooling system designed with PCM material. Because the system can reject the heat from the PCM that changes phase over a small temperature range, the PCM can minimize the temperature variation of the battery module [42].

At the same time, it is possible to start from the high-temperature resistance of the electrode, for example, by selecting electrodes that are more resistant to high temperatures. The following is an introduction to a safe, efficient, and high-temperature-resistant cathode material for Na-S batteries.

**Figure 3:**
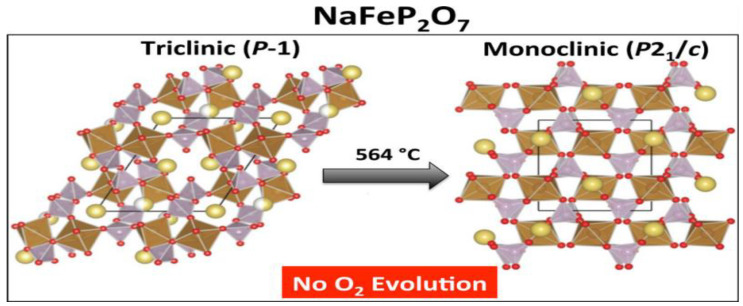
Phase transition from triclinic (P-1) to the ground state monoclinic (P2_1_/c) polymorph above 560 °C of NaFeP_2_O_7_. Reproduced with permission [43], Copyright 2013, American Chemical Society.

Na_2_FeP_2_O_7_ is a safe cathode with high thermal stability (Figure 3). High-temperature analyses of the desodiated state NaFeP_2_O_7_ show an irreversible phase transition from the triclinic (P-1) to the ground state monoclinic (P2_1_/c) polymorph above 560 °C. It demonstrates high thermal stability, with no thermal decomposition and/or oxygen evolution until 600 °C. This strongly indicates that NaFeP_2_O_7_ is an excellent material for the safe cathode of Na-S batteries [43].

## 3. Na-S Batteries at Room Temperature

### 3.1. Reaction Mechanism

**Figure 4:**
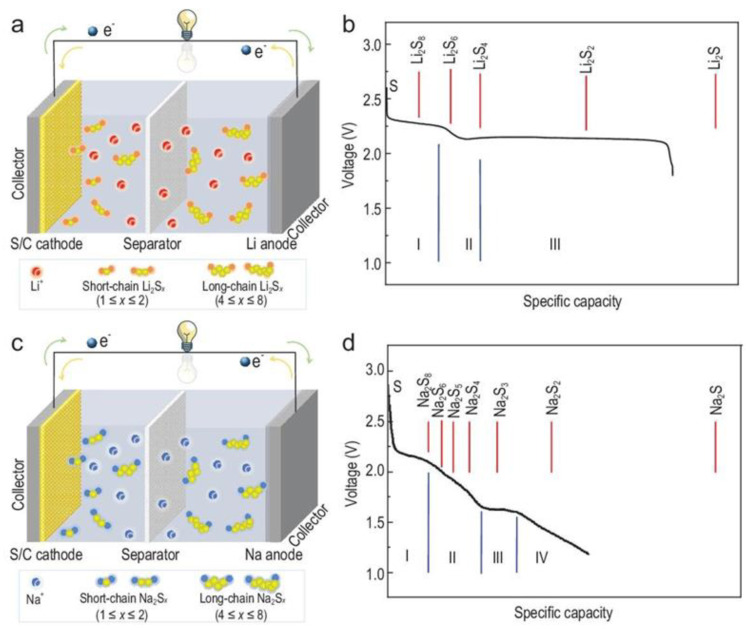
The operating principle of the RT Na-S battery vs. Li-S battery. (**a**) Schematic and (**b**) theoretical vs. practical discharge traits of a Li-S battery. (**c**) Schematic and (**d**) theoretical vs. practical discharge traits of an RT Na-S battery. Reproduced with permission [45], Copyright 2023, NSR. The Step I, II, III, IV coincide well with the Equations (1)–(6) at P7.

Figure 4a,c displayed the reaction mechanisms of RT Na-S and Li-S batteries. Both batteries possess nearly the same structure, except for divergent anodes (lithium or sodium) and analogous electrolytes (Figure 4a,c). Customarily, RT Na-S batteries consist of a metallic sodium anode, sulfur or sulfur-containing composite cathode, polymer separator and an electrolyte [44], which can be divided into liquid carbonate or ether-based electrolytes [45].

In fact, Na-S and Li-S batteries are chemically similar but not identical [46]. The theoretical capacity of the Na-S battery is 1274 Wh/kg, and the theoretical capacity of the Li-S battery is 2600 Wh/kg [47]. The multi-step reaction between the metal anode and the S cathode is mainly involved in Li-S and RT Na-S batteries, as shown in the common multi-platform phenomenon in discharge profiles (Figure 4b,d) [44]. Regarding the reaction process, ions (Na^+^ or Li^+^) resulting from the anodized oxidation of the alkali metals reach the S cathode and produce a string of intermediate products, such as soluble polysulfide Na_2_S_8_, Na_2_S_6_ and Na_2_S_4_ and baffling devices Na_2_S_3_ and Na_2_S_2_, which are finally fully converted to Na_2_S. Based on the fact that there are two electron transfers per mole, the academic definite capacity of S is extremely high, up to 1675 mA h/g, corresponding to the production of Li_2_S or Na_2_S [45].

The specific process of RT Na-S batteries reaction is as follows:

In the discharge process, the metal Na anode is oxidized to Na^+^ ions [48], as shown in Equation (1) below:

Na → Na^+^ + e^−^ (1)

At the same time, on the S cathode side, the sulfur element undergoes a series of complex reduction reactions (Figure 4d) to produce a variety of intermediates. In the high-pressure platform region I (~2.20 V), the solid-liquid reaction crops hugely dissoluble Na_2_S_8_, which is given in Formula (2):

S_8_ + 2Na^+^ + 2e^−^ → Na_2_S_8_ (2)

After that, in the inclined region II (2.20–1.65 V), the dissolved Na_2_S_8_ is converted to the dissoluble Na_2_S_4_, which is composed of a liquid-liquid reaction (Equation (3)). At this stage, Na_2_S_6_ and Na_2_S_5_ may be formed from other subtle reactions. Compared with lithium polysulfide (Na_2_S_x_, 4 ≤ x ≤ 8), the generated sodium polysulfide Na_2_S_x_, 4 ≤ x ≤ 8) is more soluble in liquid electrolytes and has a stronger shuttle effect.

Na_2_S_8_ + 2Na^+^ + 2e^−^ → 2Na_2_S_4_ (3)

The reaction corresponding to region III is the liquid-solid transformation of dissolved Na_2_S_4_ into intermediate products of insoluble Na_2_S_3_ or Na_2_S_2_ at ≈ 1.65 V (Formulas (4) and (5)).

Na_2_S_4_ + 2/3Na^+^ + 2/3e^−^ → 4/3Na_2_S_3_ (4)

Na_2_S_4_ + 2Na^+^ + 2e^−^ → 2Na_2_S_2_ (5)

Region IV (1.65–1.20 V) corresponds to a solid-solid conversion of insoluble Na_2_S_2_ to Na_2_S (Formulation (6)).

Na_2_S_2_ + 2Na^+^ + 2e^−^ → 2Na_2_S (6)

However, it is worth noting that there is no strong experimental evidence to show that the actual electrochemical reaction must follow the step-by-step process indicated by the above equation, i.e., whether the conversion from sodium polysulfide to sodium monosulfide from sulfur must be complete. In the actual electrode reaction, the intermediate product of the electrode reaction may be a mixture of several polysulfides, depending on the kinetic characteristics of the reaction reactions and the chemical stability of the reaction products. Noteworthy is that in high-temperature (300–350 °C) Na-S secondary batteries, it is broadly believed that the electrode reaction is limited to S to Na_2_S_3_, thereby its theoretical energy density is low, which is primarily caused by the characteristics of reaction intermediates (Table 2) [45].

### 3.2. Features

RT Na-S batteries reduce the operating temperature of the battery, can reduce the cost of fabricating sodium sulfur batteries [49], avoid a series of serious consequences such as fire combustion caused by high temperatures, ensure the utilization of sodium-sulfur batteries and increase their service life [3]. The following are several room-temperature Na-S batteries that have been developed so far.

#### 3.2.1. Medium Temperature Flat Sodium Battery

**Figure 5:**
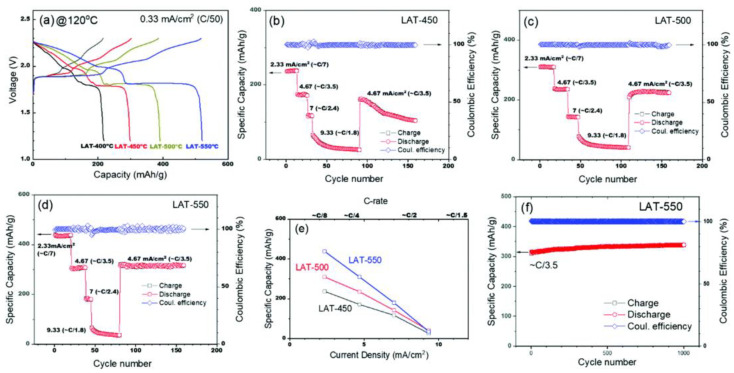
Battery performance of Na-S cells at 120 °C. (**a**) Voltage profiles of cells containing BASE treated with lead acetate trihydrate (LAT) at various temperatures. (**b**–**d**) Capacity and Coulombic efficiency for the Na-S cells containing BASE treated with LAT at 450, 500, and 550 °C, respectively. (**e**) Specific capacity retention with respect to current density (C-rate). (**f**) Stable long-term cycling (C/3.5 and 1000 cycles) performance of the Na-S cell containing BASE treated with LAT at 550 °C. Reproduced with permission [52], Copyright 2020, Royal Society of Chemistry.

This battery uses Beta alumina as a solid separator and tetraethylene glycol dimethyl ether as a positive electrolyte, which provide enough platform to enhance the solubility of S and Na_2_S_4_ (Figure 5). In addition, NaI salt can also be utilized to enhance the ionic conductivity of the electrolyte. For the goal of improving the power characteristics of the battery [50], the battery structure can be transformed from the traditional tube structure to the plate structure, where the diaphragm is a 600-μm thick Beta alumina ceramic sheet. When the battery operated at 150 °C, it displayed an original specific capacity of 450 mA h/g. Even though the obtained battery suffered from the 60-cycle test with a prevailing density of 2.23 mA/cm^2^, the capacity retention rate is still greater than 70% [51].

#### 3.2.2. Solid-State Sodium Battery

**Figure 6:**
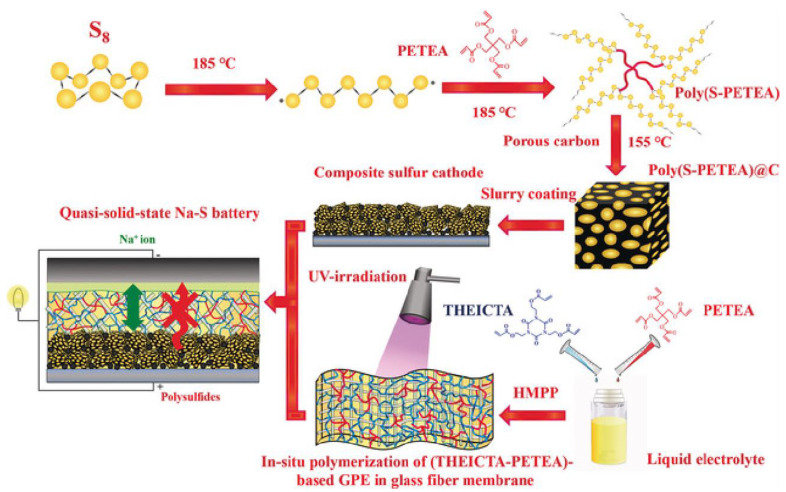
Schematic illustration of the preparation of the quasi-solid-state Na-S battery. Reproduced with permission [54], Copyright 2018, Wiley.

A solid-state sodium battery utilizes the solid metal sodium as the negative electrode, and the operating temperature is below the melting point of sodium metal [53]. Recently, the American Ceramatec company proposed a solid-state sodium battery concept system with a power module of 20–40 kWh, the size of a refrigerator, and a battery operating temperature below 90 °C (Figure 6). However, the breakthrough is that the battery modules are solid rather than hot liquid when operating at normal temperatures. Conventional sodium batteries were operated at temperatures of around 300 °C with the electrode active substance in a hot liquid state, which is highly corrosive and harmful. For comparison, the novel-state sodium battery runs at least 4 h at 5 kW and possesses an operating life of more than 10 years [51].

**Figure 7:**
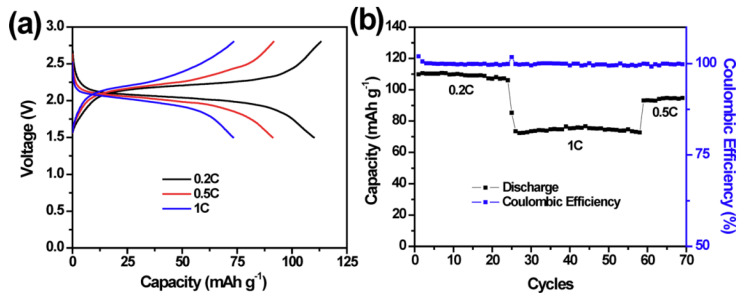
(**a**) Charge and discharge voltage profiles and (**b**) cycling performance of NaTi_2_(PO_4_)_3_/Na cell with an H-NASICON pellet electrolyte at different C-rates and 65 °C. Reproduced with permission [55], Copyright 2016, Scientific Reports.

The graph below shows the charge/discharge voltage curves for some of the solid-state sodium batteries and the cycling performance of the NaTi_2_(PO_4_)_3_/Na battery using the H-NASICON pellet electrolyte at different C-rates and 65 °C (Figure 7) [55].

#### 3.2.3. Sodium Molten Salt Battery

A sodium molten salt battery utilizes non-combustible molten salt as an electrolyte and displays the advantages of high energy density and good safety performance, which need to be heated to 90 °C to guarantee the melting of molten salt. Besides, benefiting from the high energy density and the low requirement for cooling space, miniaturization and lightweighting of battery systems will be possible in the future. Molten salt is non-combustible and non-volatile, as well as having the characteristics of high ion solubility, which render the battery can be operated at a temperature of 90 °C. Further studies have shown that these batteries have a theoretical capacity of 25–250 kWh per pack, a cell efficiency of 87%, and a service life of 2500 cycles at 100% depth of discharge and 4500 cycles at 80% depth of discharge [56]. It can also withstand a high power discharge of 15 C (50 A/cm^2^) in a short time [51].

#### 3.2.4. Aqueous Sodium-Ion Battery

**Figure 8:**
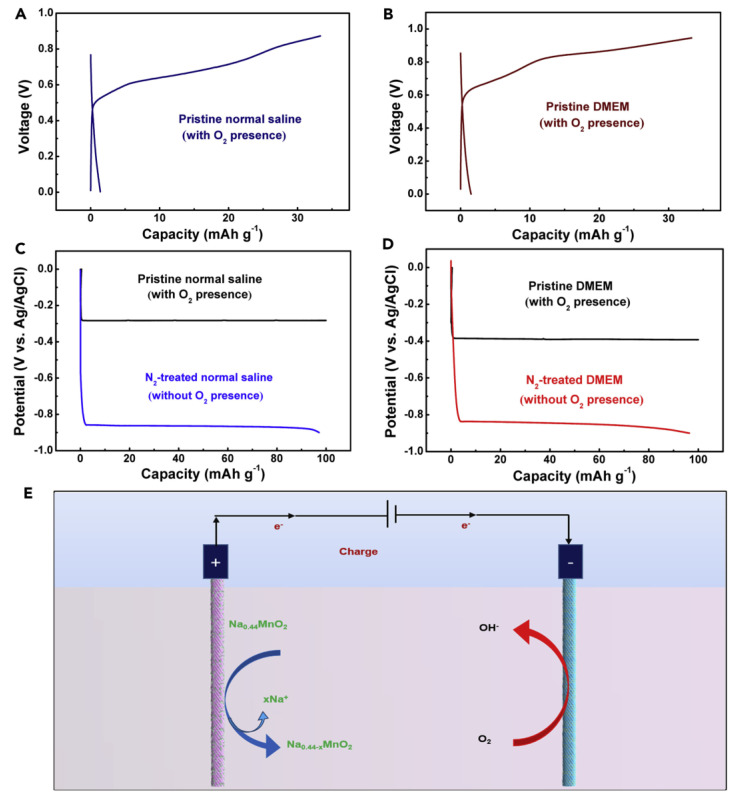
Electrochemical deoxygenation function of fiber-shaped aqueous SIBs. (**A**,**B**) Galvanostatic charge-discharge curves at a current density of 0.2 A g^−1^ of the fiber-shaped SIBs using pristine normal saline (with O_2_) (**A**) and pristine DMEM (with O_2_) (**B**) as electrolyte. The charge time was controlled at 10 min for preventing the oxygen revolution reaction; (**C**) Discharge curves (tested at a current density of 0.2 A g^−1^) of the fiber-shaped CNT/NaTi_2_(PO_4_)_3_@C hybrid electrodes in pristine normal saline solution with O_2_ (black line) and in N_2_-treated normal saline solution without O_2_ (blue line); (**D**) Discharge curves (tested at a current density of 0.2 A g^−1^) of the fiber-shaped CNT/NaTi_2_(PO_4_)_3_@C hybrid electrodes in pristine DMEM solution with O_2_ (black line) and in N_2_-treated DMEM solution without O_2_ (red line); (**E**) Schematic showing the operation mechanism of the deoxygenation function of the fiber-shaped aqueous SIB. The CNTs have catalytic activity for O_2_ electrochemical reduction, which was demonstrated in our previous report Reproduced with permission [59], Copyright 2017, Science Direct.

In recent years, the aqueous electrolyte sodium ion battery has attracted much attention from the industry. Compared with the organic solvent electrolyte sodium ion battery, the aqueous electrolyte sodium ion battery has higher safety and a lower cost. The battery typically utilized activated carbon as the negative electrode, non-woven fabric as the diaphragm and alkali metal ion intercalation compounds λ-MnO_2_ and NaTi_2_(PO_4_)_3_ as the positive electrode. All these electrode materials are non-toxic, non-combustible and free of explosive risk. Besides, the battery also displayed superior cycle stability and energy efficiency, which are very suitable for the power grid day and night peak cutting, valley filling and solar grid connection. Compared with the conventional Na-S battery (Figure 8) [57], the battery does not need high temperature maintenance [58], has non-combustible and corrosive substances, and belongs to the intrinsically safe operating battery at room temperature [51].

### 3.3. Challenges

Room-temperature Na-S batteries also face many challenges. For example, the final product of sulfur cathode discharge generates Na_2_S and expands by about 160%, which can easily cause electrode material shedding. Moreover, the intermediate product, polysulfide, will dissolve in the electrolyte and shuttle to the negative electrode for irreversible side reactions, resulting in rapid capacity decay. The sodium dendrites that result from the cycle of sodium metal negative electrodes can puncture the diaphragm and cause a short circuit [44]. Therefore, the development of stable and safe electrode materials is essential for room-temperature Na-S batteries [60].

Based on this, utilizing Na_2_S, the discharge end product of room temperature Na-S batteries [61], as the cathode not only eliminates the volume expansion problem of the sulfur cathode [22] but also provides a sodium source that can be paired with other safe cathodes (e.g., hard carbon, tin metal, etc.) to avoid the safety hazards caused by the direct use of sodium metal cathodes [62]. The shuttle effect occurs for the following reasons [63].

Despite the many advantages of Na_2_S cathode material, the current research on Na_2_S as a room temperature Na-S battery cathode material is still in its infancy, which can be attributed to poor intrinsic conductivity and slow conversion kinetics of Na_2_S and polysulfide in the charging and discharging processes [1]. Additionally, the intermediate product polysulfide will dissolve into the electrolyte, crossing to the surface of the cathode and causing the self-discharge phenomenon, resulting in the loss of active material and the rapid decay of capacity [64], i.e., the “shuttle effect”, which limits their practical application [65].

Due to their similar operating principle to lithium-sulfur batteries, room-temperature Na-S batteries are prone to suffer from the following problems: During the charging and discharging processes [66], sodium polysulfide Na_2_S_n_ (4 ≤ n ≤ 8) intermediates exhibit high solubility in organic solvents [61], and the polysulfide may be diffused from the positive electrode to the negative electrode and reduced on the negative surface. The soluble reducing products return to the positive electrode for oxidation and further induce the “shuttling effect”, leading to the sodium electrode surface deteriorating and significantly reducing battery cycle life [65].

### 3.4. Solutions

#### 3.4.1. Na_2_S/C Composites

**Figure 9:**
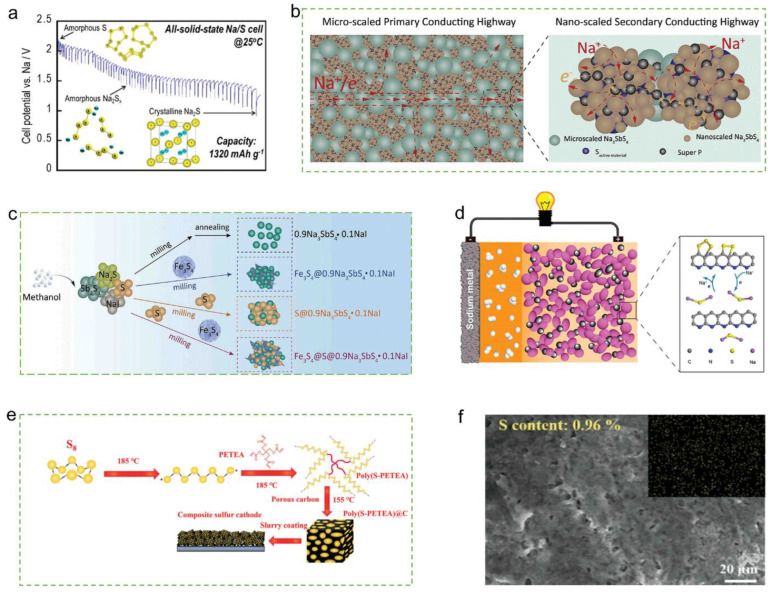
Summary of the construction of the composite sulfur cathodes. (**a**) Schematic illustration of S-AB-Na_3_PS_4_; (**b**) Illustration of the ionic/electronic conducting channels in S-Na_3_SbS_4_-C composite cathode; (**c**) Schematic illustration of Fe_3_S_4_@S@0.9Na_3_SbS_4_0.1NaI; (**d**) Schematic illustration of S/CPAN; (**e**) Schematic illustration of the preparation of the poly(S-PETEA)@C. (**f**)The FE-SEM images and corresponding elemental maps of Na/GPE/poly(S-PETEA)@C cells. Reproduced with permission [6], Copyright 2023, Wiley.

Carbon-based materials are typically utilized as conductive substrate materials to composite with Na_2_S, which can effectively improve the overall electrical conductivity of Na_2_S/C composites by taking advantage of the excellent electrical conductivity of carbon-based materials and increasing the interfacial charge transfer rate [1]. Although commercial Na_2_S can directly serve as the cathode material for room temperature Na-S batteries, it still needs to overcome a large overpotential during its first turn of charging, mainly due to the large size of commercial Na_2_S particles (micron level) and the relatively large amount of nucleation when converted to polysulfide, as well as the limited effect of direct compounding of commercial Na_2_S with carbon. Therefore, in situ preparation of Na_2_S composites with conductive materials will be favorable to further enhance the electrical conductivity of the Na_2_S composite anode and simultaneously reduce the overpotential during first-loop charging (Figure 9) [60].

#### 3.4.2. Na_2_S Morphology Modulation

Modulating the morphology of Na_2_S can also promote the diffusion of sodium ions and rapid conduction of electrons [67], thus improving the reversible capacity and cycle life of room-temperature Na-S batteries [68]. To this end, reducing the particle size of Na_2_S can reduce its activation energy barrier and thus achieve an increase in the reversible capacity of Na-S batteries [60].

**Figure 10:**
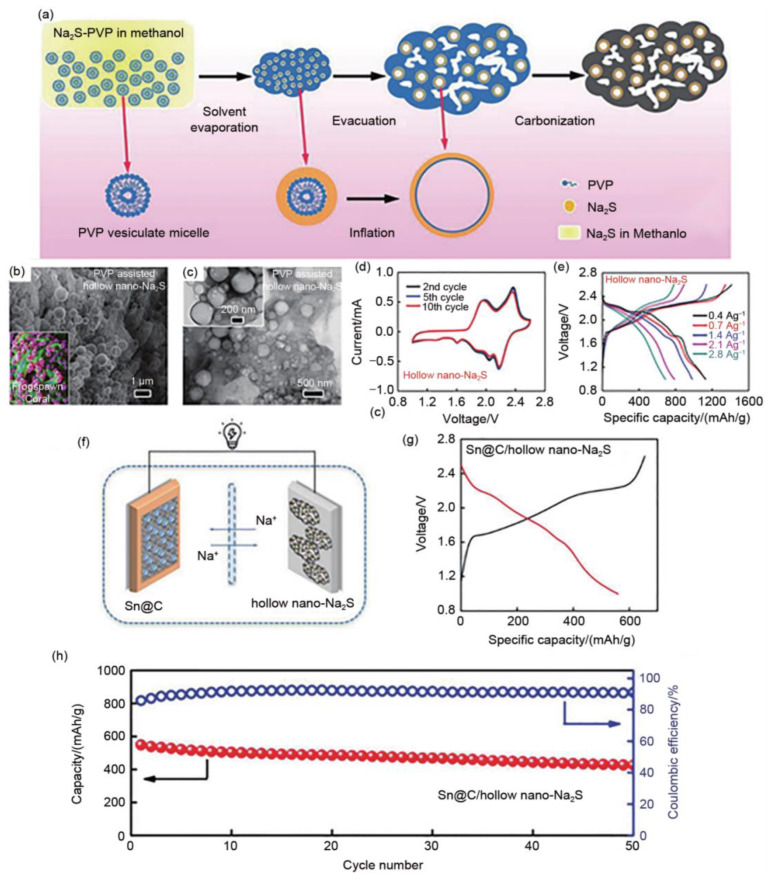
(**a**) Schematic illustration for the synthesis of commercial hollow nano-Na_2_S composite and (**b**,**c**) corresponding with its TEM images; (**d**,**e**) half-cell and (**f**–**h**) full-cell performance; (**g**) The typical galvanostatic charge-discharge voltage profile (red and black line) of Sn@C/hollow nano-Na_2_S; (**h**) Cycling performance (red line) and Coulombic efficiency (black line) of Sn@C/hollow nano-Na_2_S. Reproduced with permission [69], Copyright 2018, Wiley.

A frog-egg coral-like composite anode material with Na_2_S hollow spheres embedded in a porous conductive carbon skeleton was successfully prepared by mixing commercial Na_2_S pellets (hollow nano Na_2_S composite) with polyvinylpyrrolidone (PVP) and methanol solution after solvent evaporation, evacuation and carbonization (Figure 10) [60].

#### 3.4.3. Catalyze Reversible Cycling of Na_2_S

The low intrinsic reactivity of Na_2_S leads to slow transformation kinetics between Na_2_S and polysulfide, which further intensifies the “shuttle effect” of polysulfide [54]. Therefore, regarding the design of cathode materials, the catalyst is introduced to improve the reactivity of Na_2_S, and the reversible cycle of Na_2_S and polysulfide catalyzed by a high-efficiency catalyst can realize high efficiency and capricious room-temperature Na-S batteries [60].

**Figure 11:**
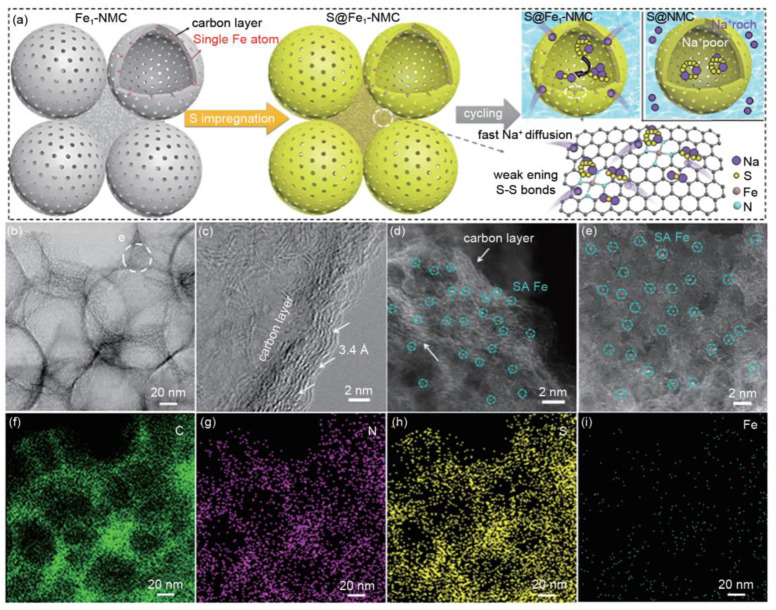
Electron microscope images of S@Fe_1_-NMC. (**a**) Schematic illustration of the synthesis of S@Fe_1_-NMC and electrode reaction mechanism of S@NMC and S@Fe_1_-NMC. (**b**–**e**) TEM images and high-resolution (HR) STEM images of S@Fe_1_-NMC. (**f**–**i**) Corresponding elemental mapping of S@Fe_1_-NMC. Reproduced with permission [70], Copyright 2021, Science Direct.

Zhang et al. reported that the introduction of atomic-scale Co catalysts in the design of S cathodes could catalyze the decomposition of the intermediate product Na_2_S_4_ and inhibit the shuttle effect of polysulfides. Meanwhile, Zhang et al. originally proposed a bifunctional mechanism for atomic Fe catalysts, using atomic Fe catalysts to disperse the sulfur cathode, weaken the S-S bond in the polysulfide, improve the reactivity of the polysulfide, accelerate the diffusion rate of sodium ions, and successfully suppress the shuttle effect. The first cycle-specific capacity of 1650 mAh/g and the current density of 100 mA/g were obtained, and the specific capacity was maintained at 540 mAh/g after 500 cycles, which opened up a new avenue for the preparation of high-performance cathode materials (Figure 11) [70].

#### 3.4.4. Battery Structure Design

**Figure 12:**
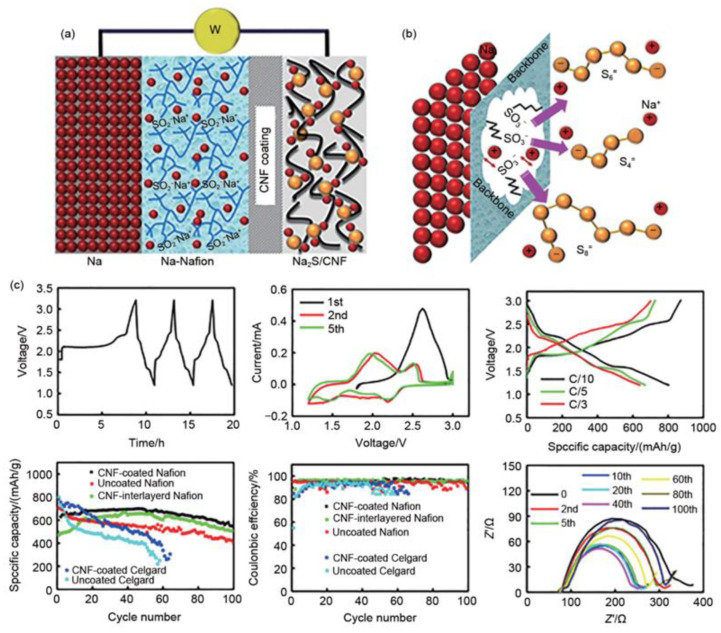
(**a**) Schematic of a Na‖Na-Nafion/AC-CNF coating‖Na_2_S/AC-CNF cell, (**b**) Schematic of the ionic selectivity of the Nafion membrane, (**c**) The electrochemical performance of Na‖Na-Nafion/AC-CNF coating‖Na_2_S/AC-CNF cell. Reproduced with permission [72], Copyright 2016, American Chemical Society.

Apart from the electrode material design, the researchers also improved the electrochemical activity of the Na_2_S cathode material by optimizing the battery structure to improve the diffusion rate of sodium ions and the “shuttle effect” of polysulfide [71]. S inhibit the transfer of polysulfide during the charging and discharging processes of the Na_2_S cathode material, the researchers developed carbon-coated membrane electrodes to replace the conventional Nafion membranes and Na_2_S positives (Figure 12a). The as-obtained membrane electrode realizes the enhanced conduction of sodium ions and only allows the diffusion of sodium ions when served as a cation-selective membrane, thus inhibiting the shuttle of polysulfide anions (Figure 12b). Moreover, the carbon-coated Nafion membrane can also act as a fluid collector to achieve high-efficiency electrochemical utilization of Na_2_S cathode materials [60].

## 4. Conclusions and Outlook

The evolution of more feasible electrodes for RT Na-S batteries will be driven by sustainable raw materials, simple processes and excellent electrochemical performance [17]. Na-S batteries can achieve a high speculative gravimetric energy density of 1274 kWh/g by combining the high theoretical definitive capacities of Na (1166 mA h/g) and S (1672 mA h/g) [73]. High-temperature Na-S (HT-Na-S) batteries working at ≈300 °C with an intellectual energy density of 760 kWh/g using Na_2_S_3_ as the final discharge product were installed at an affordable material cost. In theory, RT-Na-S batteries offer a higher energy density through the two-electron reaction of S and Na, along with Na_2_S as the final product, and the room-temperature operation reduces maintenance costs and addresses safety concerns as there is no risk of liquid Na metal leaking in HT-Na-S batteries. These advantages mean that RT-Na-S batteries are expected to be utilized to store energy in stationary applications while remaining cost effective [72].

In this work, we explore the latest research results on RT Na-S batteries by understanding their history and emphasize the importance of the study of RT Na-S batteries. Firstly, this review takes the working mechanism of the RT Na-S battery as its starting point and then goes deeper into the RT Na-S battery through specific analyses [44]. Secondly, we understand the different characteristics of Na-S batteries at different temperatures and summarize their advantages. Finally, we proposed the challenges faced by Na-S batteries at high and medium temperatures, as well as the corresponding solutions, prospects and development potential.

Although several advances have been made in RT Na-S batteries, the relatively short development history and limited knowledge mean that there is still a long way to go to achieve more stable and durable RT Na-S batteries [74]. More research must be devoted to RT Na-S batteries themselves, including electrochemical mechanisms, scalable electrode material production and practical full-cell standardization [75]. First, for electrochemical mechanisms, the application for large-scale energy storage is limited by safety aspects due to the flammability of the electrolyte and the formation of sodium dendrites during cycling. The development of a low-temperature all-inorganic solid-state Na-S battery (ASNSB), in which the inorganic solid-state electrolyte is intrinsically non-flammable, is the ultimate solution to the safety issue for Na-S batteries [76]. Second, as for electrode materials, MXene-based materials are regarded as one of the electrode materials with the greatest potential for use in sodium-ion-based devices [77]. Third, more attention will be paid to adsorption and catalytic strategies for RT Na-S chemistry, with the discussions of various adsorption strategies with a wide variety of forms and principles, including nanostructured confinement, heteroatom doping, covalent bonding and polar interactions [78]. In a word, we expect that low-cost, high-performing RT Na-S batteries will enter the market sooner, following significant research efforts, and play an important role in future grid-based energy storage systems [75].

## Figures and Tables

**Table 1:** Electrochemical performance of Na-S battery operated at a wide temperature.

Electrochemical Performance of Na-S Batteries	High Temperature	Room Temperature
**Energy density**	200 Wh/kg or higher	150~240 Wh/kg
**Cyclic lifespan**	1000~3000 times	2500~4500 times
**Power density**	2000 hundred watts per kilogram or above	

**Table 2:** Comparison of lithium-ion batteries, lithium-sulfur batteries, and Na-S batteries.

	Li Ion Batteries	Li-S Batteries	Na-S Batteries
**Energy density**	100–265 Wh/kg	200–300 Wh/kg	90–150 Wh/kg
**Cyclic lifespan**	Thousands of times more	100–1000	Thousands of times more
**Power density**	1000–3000 W/kg	Below 1000 W/kg	100–500 W/kg
**Cost**	High	Very high	Low
**Environmental protection**	Both have minimal environmental Impact, but lithium resources are limited.		It has minimal impact on the environment and abundant resources.

## Data Availability

The authors do not have permission to share data.
